# Comparison of Cleaning Efficiency of Root Canal Using Different Rotary NiTi Instrumentation System: A Scanning Electron Microscopic Study

**DOI:** 10.7759/cureus.24200

**Published:** 2022-04-17

**Authors:** Sakshi Agrawal, Anjali Bichpuriya, Rahul Maria

**Affiliations:** 1 Department of Conservative Dentistry and Endodontics, Bhabha College of Dental Sciences, Bhopal, IND

**Keywords:** f360, wave one, cleaning, ni-ti, rotary instrument, scanning electron microscopy

## Abstract

Background: To ensure that endodontic treatment is as effective as possible, it is important to remove any smear layer that forms as part of the instrumentation procedure. This layer might reduce the overall effectiveness of endodontic therapy.

Aim of the study: For this research, two distinct types of rotary NiTi files were compared: WaveOne Gold (WOG) (Dentsply Maillefer, Ballaigues, Switzerland) and F360 (Komet Brasseler GmbH & Co., Lemgo, Germany) for its capacity to eliminate trash and the smear layer.

Materials and methods: Two groups (n=20 each) of 40 mandibular second premolar teeth were employed in this investigation, with each group receiving a random allocation of teeth. The F360 system and the WaveOne Gold system are two sets of instruments. The samples were irrigated with a mixture of sodium hypochlorite (NaOCl) (5.25%) and citric acid (40%). Finally, all samples in the centre of the coronal, middle, and apical thirds were examined by scanning electron microscopy (SEM). Mann-Whitney U tests were used to analyse the data.

Results: F360 instrument showed a statistically significant difference for smear layer removal among all thirds of the root canal whereas WOG resulted in a significant difference when the apical third was compared to the middle and coronal third. Significant differences were found in the middle and apical third in terms of smear layer removal between the two groups.

Both F360 and WOG instruments showed statistically significant differences for debris removal among all thirds of the root canal. No significant differences were found in the coronal, middle, and apical third in terms of debris removal between the two groups.

Conclusion: WOG resulted in cleaner canals compared to the F360 file system at coronal, middle, and apical third.

## Introduction

Root canal therapy aims to eliminate intracanal bacteria, which are the primary cause of infection. Effective endodontic treatment requires a thorough chemo-mechanical preparation [1].

The most successful root canal treatment may be obtained if the root canal system is thoroughly cleaned, shaped, and sealed [2]. Root canals cannot be cleaned reliably with current equipment, particularly in the apical area [3]. For cleaning purposes, just a superficial removal of pulp tissue was performed on the canals using stainless steel tools. Stainless steel files have also been shown to cause canal aberrations, such as ledges, holes, zippers, and elbows [4]. Some of the shortcomings of conventional endodontic instruments have been addressed with the development of nickel-titanium (NiTi) instruments [1].

Because of the relatively recent development of rotary NiTi tools, it is now possible to quickly and easily prepare canals with a constant taper [5]. Scratches appear on every file that may be opened, and this is particularly true in the uppermost region of the root canal system because of its limited ability to clean [6].

This variance in the efficacy of debris removal of NiTi instruments may be due to flute design variations, as shown by several studies. Rotary NiTi instruments with improved blade designs have recently been introduced to improve root canal preparation efficiency [7]. Rotational dentistry nowadays causes a thick coating of a dental debris and smear layer to coat the canal walls [8]. Remaining pulp tissue and germs that have remained on the dentinal walls after mechanical root canal instrumentation are contained inside this 1 to 2 um thick layer of dentin debris [9]. Failures in root canal therapy may be due to the existence of a smear layer and debris, particularly at their apices, which may include bacteria and other microorganisms that cannot thrive in the root canal's environment [7].

The root canal disinfection process relies on irrigation solutions, which are considered critical to the treatment's effectiveness. To make mechanical preparation easier, they are also necessary for the debridement of the canals [10]. Most people use sodium hypochlorite (NaOCl) as an irrigant because it is an excellent antibacterial, but it does not remove the smear layer from the dentin walls like other methods [11]. Ethylenediaminetetraacetic acid (EDTA) is regarded as a modest antibacterial agent, but its ability to chelate hard tissue as a decalcifying agent has made it popular [2].

Continuous motion or reciprocating motion NiTi tools have been used to further enhance and ease mechanical root canal preparations [12].

Thus, research was conducted to assess the cleaning effectiveness of the F360 and WaveOne Gold (WOG) rotary file systems.

## Materials and methods

The study selection was based on the following:

Inclusion criteria

1. Freshly extracted non-carious tooth. 2. Single-rooted mandibular premolar teeth with intact, fully formed apices

Exclusion criteria

1. Fractured tooth. 2. Resorptive defects or calcifications in the tooth.

Study design

A scaler to remove calculus and soft tissue debris from each tooth then rinsed with tap water and placed in a distilled water solution to maintain their cleanliness. The decision was made to keep the crowns in place so that more irrigating solutions could reach the canal walls. A standardized access cavity was made for each sample used in the study. After negotiating each root canal using a #10 stainless steel K-file, the working length was determined by subtracting 0.5 mm from the actual canal length of each one.

These file systems were chosen due to both file systems being a single file system, to know the difference between continuous and reciprocation, to know the difference between constant and variable tapers in cleaning efficiency, to know the difference between 4% and 7% taper in cleaning efficiency, to know the difference between WOG heat-treated, F360 conventional. 

As a result, the samples were separated into two equal groups by lot (n=20). Group 1: Samples were instrumented with the F360 system (25/0.4). Group 2: Samples were instrumented with WOG (25/0.7).

Each sample was irrigated with 5ml of 5.25% NaOCl, and 17% EDTA with a final flush of 1ml of 40% citric acid, which was activated using an endoactivator. The irrigation solutions were introduced in the canals utilizing NaviTip®31ga Double Sideport Irrigator Tip (21 mm, yellow) (Ultradent, USA). For each of the systems used in the research, the samples were instrumented in accordance with the manufacturer's guidelines.

Data collection

All teeth were shaped and sectioned buccolingually. Scanning electron microscopes (JEOL JSM 6000 Plus, 1000X magnification) were utilized to examine the gold-sputtered samples. An SEM, which was used to evaluate the result of the study, has been shown to offer high-definition three-dimensional images with superior magnification and resolution. The final grade was based on a numerical scale that was employed. (Tables 1, 2) 

**Table 1:** Debris score criteria

Debris score
Score 1: Root canal walls that are free of even the tiniest pieces of detritus.
Score 2: Few large clusters of trash.
Score 3: Approximately 50% of the root canal wall is covered by a thick layer of debris.
Score 4: In more than half of the root canals, there was a layer of debris.
Score 5: Debris covers the whole or almost the entire root canal wall.

**Table 2:** Residual smear layer score

Smear layer score
Score 1: Dentinal tubules were completely free of any smear layer.
Score 2: One or two dentinal tubules were exposed, as well as some smear layer.
Score 3: Few dentinal tubules are exposed due to a uniform smear on the root canal's wall.
Score 4: A uniform smear layer covered the whole root canal wall, and no exposed dentinal tubules could be seen.
Score 5: Root canal walls are covered with a thick, homogenous smear layer

Statistical analysis

Analyses of smear layer and debris in canals' apical thirds were conducted using Mann-Whitney U tests for each of the three canals investigated. Kappa variant was not used in the study. 

## Results

The use of the F360 instrument showed a statistically significant difference for smear layer removal at the coronal third when compared to the middle (p-value = 0.017) and apical third (p-value = 0.001). A statistically significant difference was found between the middle and apical third of the root canal (p-value = 0.004) (Table 3).

**Table 3:** Statistical comparisons of smear layer removal at different level of the root canal in F360

F360 Smear Layer		Mean Difference	P-value	Significance
Coronal	Middle	2.05	0.017	Significant
	Apical	2.50	0.001	Significant
Middle	Coronal	1.65	0.017	Significant
	Apical	2.50	0.004	Significant
Apical	Coronal	1.65	0.001	Significant
	Middle	2.05	0.004	Significant

The use of the WOG instrument resulted in no statistically significant difference for smear layer removal when the coronal third was compared to the middle third (p-value = 0.163). A statistically significant difference was found when the apical third was compared to the middle and coronal third (p-value = 0.000) (Table 4).

**Table 4:** Statistical comparisons of smear layer removal at different level of the root canal in WOG

WOG Smear		Mean Difference	P-value	Significance
Coronal	Middle	1.40	0.163	Not Significant
	Apical	1.95	0.000	Significant
Middle	Coronal	1.20	0.163	Not Significant
	Apical	1.95	0.000	Significant
Apical	Coronal	1.20	0.000	Significant
	Middle	1.40	0.000	Significant

The use of the F360 instrument showed a statistically significant difference for debris removal at the coronal third when compared to the middle (p-value = 0.010). A statistically significant difference was found when the apical third was compared to the middle and coronal third (p-value = 0.000) (Table 5).

**Table 5:** Statistical comparisons of residual debris removal at different level of the root canal by F360

F360 Debris		Mean Difference	P-value	Significance
Coronal	Middle	1.80	0.010	Significant
	Apical	2.90	0.000	Significant
Middle	Coronal	1.50	0.010	Significant
	Apical	2.90	0.000	Significant
Apical	Coronal	1.50	0.000	Significant
	Middle	1.80	0.000	Significant

The use of the WOG instrument showed a statistically significant difference for debris removal at the coronal third when compared to the middle (p-value = 0.016). A statistically significant difference was found when the apical third was compared to the middle and coronal third (p-value = 0.000) (Table 6).

**Table 6:** Statistical comparisons of residual debris removal at different level of the root canal by WOG

WOG Debris		Mean Difference	P-value	Significance
Coronal	Middle	1.75	0.016	Significant
	Apical	2.60	0.000	Significant
Middle	Coronal	1.30	0.016	Significant
	Apical	2.60	0.000	Significant
Apical	Coronal	1.30	0.000	Significant
	Middle	1.75	0.000	Significant

The use of the WOG instrument resulted in significantly higher smear layer removal compared to F360. Both the rotary file systems showed effective cleaning in the coronal third followed by middle thirds with maximum smear layer at apical thirds of the root canal. Significant differences were found in the middle (p-value = 0.006) and an apical third (p-value = 0.045) in terms of smear layer removal between the two groups. (Table 7)

**Table 7:** Mann–Whitney tests, for intergroup comparison of the smear layer removal for establishing which file system has better cleaning efficiency

Group Comparison Smear layer		Mean Difference	P-value	Significance
Coronal	F360	1.65	0.058	Not Significant
	WOG	1.20		
Middle	F360	2.05	0.006	Significant
	WOG	1.40		
Apical	F360	2.50	0.045	Significant
	WOG	1.95		

The use of the WOG instrument resulted in significantly higher debris removal when compared to F360. Both the rotary file systems showed effective cleaning in the coronal third followed by middle thirds with maximum debris at apical thirds of the root canal. No significant differences were found at the coronal (p-value = 0.163) middle (p-value = 0.772) and apical third (p-value = 0.267) in terms of debris removal between the two groups. (Table 8)

**Table 8:** Mann–Whitney tests, for intergroup comparison of the debris removal for establishing which file system has better cleaning efficiency

Group Comparison Residual debris		Mean Difference	P-value	Significance
Coronal	F360	1.50	0.163	Not Significant
	WOG	1.30		
Middle	F360	1.80	0.772	Not Significant
	WOG	1.75		
Apical	F360	2.90	0.267	Not Significant
	WOG	2.60		

## Discussion

When performing the endodontic treatment, the root canal system must be shaped and cleaned to eliminate necrotic pulp tissue, diseased dentine, and debris so that the periapical healing process may begin [13]. Using rotating NiTi devices such as the F360 and WOG, some of these objectives were put to the test in vitro in this work. We do not have a lot of information yet on how well these two tools clean natural teeth.

Instrument cleaning efficiency was evaluated in this research based on two different parameters: debris and smear layer (or film). For the most part, the root canal wall is infected with dentine chips and pulp tissue that has adhered to the root canal wall. As a consequence, the root canal system may be unable to adequately eliminate bacteria. A buildup of debris might obstruct the sealing of the root canal, making it impossible for it to be entirely obturated [14].

To describe an inorganic, thin layer that occurs after a canal has been instrumented (by an endodontist, for example), we might refer to it as the "smear layer”. Remove the smear layer because it might prevent irrigants or antibacterial medications from reaching the dentinal tubules and so jeopardise the complete closure of a root canal [6].

“Debris, as well as smear layer, may be removed by using antibacterial irrigants in conjunction with chelating agents.”

A mixture of sodium hypochlorite, EDTA, and citric acid was utilised in this experiment. Because EDTA-containing chelating specialists and citrus extracts have recently been shown to have some role in effective trench divider cleanup following NiTi instrumentation, it should be considered that the two instruments evaluated in this study could be further improved by using a combination of NaOCl, EDTA, and citric acid [15].

In the present study, it was observed that the WOG instrumentation group showed a more cleaning ability than F360 for smear and debris removal. The result of our study can be compared to a study done by Dagna A et al. [8], who reported that F6 Skytaper was more effective than F360. The reason may be because of the constant and less taper of the F360 file 0.4%, thus reducing the effective shaping and debridement of the root canal by irrigation solution in coronal middle and apical third whereas WOG 0.7% increasing taper provides more flow of irrigation in conjunction with agitation into the root canal system. This result can be compared to a study done by Usman N et al. [3], who showed that increased size of canal instrumentation at working length produced an increase in canal cleanliness.

An SEM, which was used to evaluate the result of the study, has been shown to offer high-definition three-dimensional images with superior magnification and resolution. Various methods have been assessed to evaluate the cleaning efficiency of root canal based on debris and smear layer, including histological sections, scanning electron microscope, confocal laser scanning microscope, atomic force microscope, optical stereo microscope, light microscope, etc.

Another study was done by Suparna SG et al. [16], which also stated that WOG files resulted in cleaner canals than other file systems. This may be due to a decrease in the taper of the WOG file system, where it has 0.7% D1-D3 taper in apical and 0.6% taper in middle and apical third 0.3%. Additionally, the file also has centered off rotation between D1 and D3 and offset of mass of rotation between D4-D16 and parallelogram cross-section. These design features minimize the contact between the file and the dentin, which results in a reduction in the probability of lateral compaction of debris and smear layer in the coronal, middle, and apical third of the root canal system [17].

The result of our study was also in accordance with the study done by Choudhary D, who showed that single-file WOG showed the highest cleaning efficacy, followed by F360 [18].

The result of our study was also in accordance with the study done by Bartols et al. [19], who reported that the Reciproc Blue file can be used to prepare the root canals to full working length in 95.6% of cases. Another study done by De-Deus et al. [20] also stated that Reciproc instruments can reach full working length in 90.7%-96.4% of cases of lower molars. This can be related to our study where WOG showed better cleaning efficiency than F360 in apical third because the reason that WOG instruments can reach full working length in 90% of cases. The reason behind this may be attributed to the thermal treatment used in the manufacturing process of WOG. The heat treatment modifies the molecular structure and provides more strength and flexibility to the file system [17].

The result of our study was also in accordance with the study done by De Carvalho et al. [21], who stated that the application of reciprocating motion during instrumentation did not result in increased debris when compared with continuous rotation motion. Another study was done by Al-Khafaji et al. [22] also stated that WOG showed the best performance in smear layer removal in comparison to other instruments. The cleaning efficiency of WOG is more effective than F360 due to different kinematics of rotary files where WOG is in reciprocating motion and F360 is in continuous motion. The limitations of the study included discrepancies in observers' agreement. And hence cleaning efficiency of WOG files in combination with newer irrigating devices could be assessed in the future. Further studies shall be performed to verify if similar results can be attained with naturally occurring mixed biofilms and in curved root canals with complex anatomy. 

## Conclusions

When using WaveOne Gold instruments in this investigation, the cleaning efficiency was much greater than using F360 equipment. The apical three-quarters of the root canal had greater debris and smear when using either of the rotary file procedures. Significant variations in the middle and apical thirds (both p > 0.05) were found between the two sets of smears, but in the remaining detritus (both p > 0.05), no alterations were seen. The use of various rotary NiTi File systems has been useful for a variety of reasons, and hence in-vivo research from this point of view can be carried out in the future. This can be useful for better endodontics cleaning and shaping, which results in proper removal of smears and debris from the canals and ultimately a good root canal therapy.
